# A New Method of Treating Uterine Hemorrhage

**Published:** 1882-10-20

**Authors:** M. R. Barker

**Affiliations:** New Castle, Penn.


### A NEW METHOD OF TREATING UTERINE HEMORRHAGE.

BY M. R. BARKER, M. D., OF NEW CASTLE, PA.

Physicians of experience know the extreme difficulty, sometimes, of arresting uterine hemorrhage, the uterus seeming to defy all remedies, and pouring forth the sanguineous fluid in spite of astringents, ergot, the tampon, etc., etc. The danger of uterine injections often causes physicians to hesitate long before resorting to them. I have been frequently placed in this dilemma, and although I have used injections successfully, yet I must admit that I always felt relieved when I found that the injection had not produced any dangerous consequences.

In view of the foregoing considerations I concluded to take a departure from the usual methods, as will be seen in the report of the following cases:

CASE I. During the latter part of October, 1881, I was called to see Mrs. H, who was suffering from uterine hemorrhage. I treated her with the ordinary remedies, but without producing any permanent benefit. The tampon, applied again and again, would, of course, produce temporary suspension of the flux, but upon its removal the hemorrhage would return as badly as ever. The constitutional effects resulting from the loss of so much blood becoming alarming, I concluded something must be done.

At my next visit I took my uterine applicator and placed upon it a spiral slide, leaving about three inches of the point uncovered. The point I wrapped with absorbent cotton in the same manner as for treating the uterine cavity for endometritis, with the exception that the end next the slide I made considerably thicker than the rest, and tied a thread around it, so as to withdraw the cotton when its purpose was accomplished. I then dipped the cotton into glycerine, and dusted it effectually with powdered persulphate of iron. I then exposed the os with a speculum, and passed my loaded applicator into the uterine cavity, and with the slide pushed off the cotton and left it. From that time my occupation in this case was gone. She made a rapid recovery.

CASE II. March 30th, 1882, was called to see Mrs. W., who had been suffering with uterine hemorrhage for two months. I was of the opinion that she had had an abortion, but she denied it. She had been under the care of another physician, who had treated her with the usual remedies, the principal one being ergot. Her clothing and bed showed very plainly the severity of the hemorrhage. I also found several clots in the vagina. I used the persulphate in the same way as in Case No. 1. When leaving I requested her to let me know in a few days how she got along. In about a week I received a note from her saying that the hemorrhage ceased from the time of the application, and that there had been no return. I have met her several times since, and she was quite well.

CASE III. Mrs. R. aborted about May 1st, 1882. I saw her June 29th. The attending physician had gone through the usual routine of remedies, administered internally, apparently without benefit. I immediately made an application of the persulphate of iron after the same manner as described in the former cases, and the result was an immediate arrest of the hemorrhage. She was in my office to-day, and as I had this article partly written, I asked her the particulars of her case. She said she had "wasted" for eight weeks, and that she had taken ergot nearly all of that time; and also that there was scarcely any discharge perceptible after I introduced the tent, and that there had been none since, beyond her periods.

It seems to me that this is a much more rational method of treating uterine hemorrhage than by either internal remedies or by injections. That it is much safer than by injections no one can doubt, and I would not hesitate now to depend upon it, to the exclusion of all other remedies. This preparation is used *ad libitum* in gynecological surgery, without injury, and I am satisfied that it is equally as beneficial and harmless in ordinary uterine hemorrhage.

I am of the openion that the benefit derived from this plan of treatment is not altogether from the styptic action of the iron, but that a portion of it arises from the intolerance of the uterus to any foreign substance in its cavity, the muscular fibres contracting forcibly to expel the intruder, and I cannot see why an ordinary case of uterine hemorrhage could not be treated successfully by non-medicated tents alone.—*Med. and Surg Rep.*

